# Fungal symbiosis alters non-host, community-level plant trait response to N enrichment in a low-nutrient sand dune system

**DOI:** 10.1007/s00442-026-05914-5

**Published:** 2026-05-31

**Authors:** Shannon L. Walker, Sarah M. Emery

**Affiliations:** Department of Biological Sciences and Center for Great Lakes and Watershed Studies, Bowling Green State University, Bowling Green, 43402 OH USA

**Keywords:** *Epichloë*, Community-weighted mean, Functional traits, Trait diversity, Dune

## Abstract

Atmospheric nitrogen (N) enrichment is known to alter plant community trait composition and diversity. Additional research in low-resource environments is needed, however, as plant communities in these systems may respond differently than those where N is less limiting. Further, fungal endophytes, such as the *Epichloë* group, alter key mechanisms of species coexistence but have yet to be studied for their effects on community-level functional traits under conditions of global change. We studied the effects of N enrichment and the presence of aboveground fungal endophyte *Epichloë amarillans* (*Epichloë* hereafter) within the dominant grass species, *Ammophila breviligulata* (*Ammophila* hereafter), on community-weighted mean (CWM) traits and trait diversity of colonizing plant species in a long-term experiment of a low-resource coastal dune system. N enrichment at both real-world and high levels increased CWMs of plant size and specific leaf area (SLA) of colonizing species, and high N enrichment initially suppressed trait diversity (FDis). While N enrichment reduced community-level specific root length (SRL), this effect disappeared when *Epichloë* was present, indicating a predominant role of the fungal endophyte. We found that N enrichment may alter plant functional traits, even in a low-resource habitat, which may increase productivity, alter erosional dynamics, and affect belowground functioning. The presence of a fungal endophyte, however, altered community response to N enrichment treatments in a key root trait indicating that its presence may affect community-level traits and belowground functioning beyond its host species.

**Supplementary Information:**

The online version contains supplementary material available at 10.1007/s00442-026-05914-5.

## Introduction

Anthropogenic nitrogen (N) enrichment is a major global change factor that has substantial effects on plant communities (Cleland and Harpole 2010). Atmospheric N deposition is one of the dominant processes distributing anthropogenic N globally and represents a substantial proportion of anthropogenic N inputs to ecosystems (Galloway et al. 2008; Decina et al. 2019). Over the past 20 years, studies involving N enrichment, often at very high levels, have shown decreases in plant species richness and taxonomic diversity (Stevens et al. 2004; Suding et al. 2005; Garces et al. 2023), increases in community productivity (LeBauer and Treseder 2008; Xu et al. 2018), and altered patterns of species and functional dominance by favoring more competitive strategies (Craine et al. 2001; Suding et al. 2005; Collins et al. 2008). These more competitive, resource-acquisitive species are characterized by traits including greater total biomass, productivity, height, and relative growth rates as well as lower-density, shorter-lived tissues (Li et al. 2015; Xu et al. 2018; Weigelt et al. 2021; Guo et al. 2022). Changes in plant community diversity and composition with N enrichment then shift community mean traits and decrease functional trait diversity (Suding et al. 2005; Wang et al. 2022), which in turn alter ecosystem functioning (Loreau et al. 2001; Xu et al. 2018). However, additional studies of N enrichment effects in low-resource habitats, where plant communities are characterized by species with conservative functional traits that may have limited responses to N enrichment (Grime 1977; Chapin et al. 1986; Suding et al. 2005; Cleland and Harpole 2010), are necessary to evaluate whether mechanisms and trait effects are comparable across diverse habitat types. Additionally, while previous studies have provided substantial insights into the underlying mechanisms of N limitation by using high levels of N, studies incorporating realistic levels of N enrichment across ecosystem types are necessary to refine theories and predict real-world effects (Craine et al. 2001; Loreau et al. 2001; Wilcots et al. 2022).

The effects of N enrichment on communities can also be mediated by microbial symbionts, especially in low nutrient systems where plants often rely on their microbial symbionts for obtaining N (Suding et al. 2005; Cleland and Harpole 2010; Kivlin et al. 2013; Wang et al. 2022; Guo et al. 2022). For example, N enrichment has been shown to reduce the abundance of legumes, which form associations with N-fixing bacteria (Suding et al. 2005), as well as the abundance of grasses that rely more heavily on arbuscular mycorrhizal fungi (AMF; Wang et al. 2022), due in part to the reduced benefits of the trade-offs involved in maintaining these symbioses (Kiers et al. 2010). Fungal endophytes—fungi living within heathy plant tissues that do not induce symptoms of disease—are arguably some of the most ubiquitous plant-fungal symbioses (Arnold, 2007; Friesen et al. 2011; Harrison and Griffin 2020). They can substantially influence host plant traits and fitness including drought tolerance, productivity, and heavy metal tolerance (Clay and Schardl 2002) and may have substantial impacts on N dynamics in ecosystems (e.g., Kivlin et al. 2013; Griffin et al. 2017; Christian et al. 2019; Harrison and Griffin 2020). The *Epichloë* group of systemic fungal endophytes (Clavicipitaceae) are vertically transmitted endophytes that infect the leaves and stems of many dominant grassland plant species (e.g., tall fescue, *Lolium arundinaceum*) and have substantial effects on host plant traits and ecosystem processes (Clay and Schardl 2002). For example, the presence of *Epichloë* has been shown to increase host plant productivity and competitiveness (Leuchtmann 1993; Clay and Schardl 2002; Saikkonen et al. 2006) and impact soil microbial community diversity, nutrient cycling, and patterns of herbivory (Marks et al. 1991; Clay and Schardl 2002; Chen et al. 2022), all of which can affect plant community and ecosystem processes (van der Heijden et al. 2008; Feng et al. 2009; Lau and Lennon 2011; Adler et al. 2013; Kraft et al. 2015; Nizamani et al. 2024). Further, *Epichloë* is often found in association with strongly dominant grass species (Leuchtmann 1993; Clay and Schardl 2002; Saikkonen et al. 2006); as dominant species often have a disproportionate effect on plant community processes (Grime 1998; Avolio et al. 2019), the potential for *Epichloë*-mediated effects on plant communities may be even greater when it is present in a strongly dominant host plant. The effects of *Epichloë* are also modulated by the availability of N and other limiting resources (Malinowski and Belesky 2000; Clay and Schardl 2002), indicating that endophyte effects on plant communities may interact significantly with anthropogenic N enrichment. Though *Epichloë* endophytes have substantial impacts on key plant community processes and associate with strongly dominant plants, studies of endophyte effects at the plant community-level are generally lacking, especially in the context of global change.

The primary goal of our study was to evaluate community responses to realistic levels of N enrichment in a low-nutrient system, as manipulated N levels in many existing studies are orders of magnitude greater than those experienced by plant communities in the real world (Ackerman et al. 2019; Decina et al. 2019; Midolo et al. 2019; Wilcots et al. 2022). We also investigated whether the presence of an *Epichloë* fungal endophyte within a dominant grass species interacted with N enrichment to drive changes in plant community functional trait composition and trait diversity of non-host plant species. We focused our study on community-level functional traits as trait-based investigations may lead to insights that are more generalizable to other systems (Díaz and Cabido 2001; Suding et al. 2008; Adler et al. 2013; Westoby 2024) and ecosystem processes can often be better predicted by functional traits and functional diversity than taxonomic species richness (Díaz and Cabido 2001; Funk et al. 2017; Xu et al. 2018). Specifically, our current work builds on previous investigations of plant community diversity and compositional response (see Garces et al. 2023) by using species mean traits to understand plant community responses to N enrichment and endophyte presence beyond taxonomic change.

To address these goals, we utilized an in-situ long-term experiment in a sand dune ecosystem to examine how chronic N enrichment, representative of real-world atmospheric N deposition, and the presence of *Epichloë amarillans* (*Epichloë* hereafter) in the dominant plant species *Ammophila breviligulata* (American beachgrass, *Ammophila* hereafter) affected community-level functional traits of colonizing species over five years in a low nutrient ecosystem (Ehrenfeld 1990; Lichter 1998). We hypothesized that (1) N enrichment would decrease functional trait diversity and shift community-level traits toward more competitive/acquisitive growth strategies, as previous work in this dune system showed that N enrichment reduced community evenness and increased the dominance of grasses (Garces et al. 2023). Alternatively, N enrichment may have little or no effect on community functional traits if plants are adapted to the very low nutrient conditions of sand dunes. As discussed above, *Epichloë* often promotes the competitiveness of its host plant and has been shown to promote more competitive features in *Ammophila*, such as smaller tillers with greater SLA and greater abundance via increased tillering (Garces et al. 2023; Walker et al. 2025), which has been shown to increase total *Ammophila* biomass under conditions of low N enrichment (Walker et al. 2025). As such, we hypothesized that (2) the presence of *Epichloë* would also promote more competitive/acquisitive strategies at the community level via competition with *Ammophila*, reducing functional trait diversity and shifting plant community mean trait composition. Alternatively, the presence of *Epichloë* may, instead, promote alternative plant strategies, thus having limited effects on community-level trait means while increasing functional trait diversity. We also hypothesized that (3) there would be a synergistic interaction between N enrichment and *Epichloë* presence on plant community traits due to both competition with *Epichloë*-infected *Ammophila* and a shift in community-level traits due to N enrichment. Finally, while not the focus of this study, we also expected that community-level functional traits would shift across time due to dune plant succession and interannual variability (Cowles 1899; Grime 1977; Ehrenfeld 1990; Lichter 1998; Stallins 2006).

## Materials and methods

### Experimental set-up

Plant samples used in this study were collected from an in-situ, long-term experiment established in Leelanau State Park, Michigan, USA (45° 10.964’, -85° 34.578’) in 2010. For a full description of the experiment, please refer to Emery et al. (2015). Briefly, ninety 2m × 2m plots were established in a dune blowout in a 2 × 3 factorial experiment designed to examine the effects of precipitation and *Epichloë* on *Ammophila* and key ecosystem processes. We manipulated endophyte presence by growing *Ammophila* plants from endophyte-free seeds and introducing *Epichloë* hyphae into a small wound in the meristem of 3–5 day old seedlings with a sterile needle. Sham-inoculated seedlings were wounded in a similar manner but did not receive any hyphae (see Emery et al. 2015). Half of the plots were planted with *Ammophila* that had been inoculated with the *Epichloë* fungal endophyte and the other half planted with *Ammophila* which had been sham-inoculated. The plots were randomly established and planted at a density of 25 tillers per plot. In 2015, precipitation treatments were removed due to lack of an effect, and in 2016 (six years following *Ammophila* establishment) N enrichment treatments were established in a subset of sham- and *Epichloë*-inoculated plots (*n* = 10 plots per *Epichloë* x N enrichment treatment group). N enrichment treatments received polymer-coated, slow-release urea fertilizer (ESN; Nutrient Ltd., New Madrid MO) which was applied seasonally (May and July) from 2016 to 2021. Fidelity of the *Epichloë* treatments was verified in 2019 using commercial immunoblot kits (Phytoscreen: Agrinostics, Watkinsville, GA) and microscopy of stained leaf material—91% of tillers from *Epichloë*-inoculated plots showed endophyte presence while 89% of tillers from sham-inoculated plots remained endophyte-free (Garces et al. 2023). Treatment groups consisted of a low N enrichment treatment representative of atmospheric N deposition around the area of Chicago, IL, USA (0.5 g NH_4_^+^/m^2^), a high N enrichment treatment intended to release plants from N limitation (10 g NH_4_^+^/m^2^), and controls which received no fertilizer.

### Field sampling and plant processing

Abundance of each plant species was recorded in each plot of the N-enrichment experiment (*n* = 60) once per year during the middle of the growing season (June-July) from 2017 to 2021. Abundance was recorded as the number of individuals using the following criteria: the number of individual basal stems for forbs and woody plants, the number of tillers for most grasses, and the number of clonal clumps for *Schizachyrium* due to the high density of each clonal unit. Total species richness across all years was 13, typical of these low-diversity dune systems (Garces et al. 2023). From these 13 species, in 2021 we focused on abundant grass (*Calamovilfa longifolia*, *Elymus lanceolatus*, and *Schizachyrium scoparium*) and forb species (*Artemisia campestris*, *Campanula rotundifolia*, *Solidago nemoralis*, and *Lithospermum caroliniense*) for analysis. Five individuals of each of these plant species were collected from control plots within the experiment using a bulb corer to a depth of 10.5 cm. Plants were kept cool in the field and then frozen until further analysis (following methods in Walker and Zinnert 2022). We excluded rare and endangered species from analyses due to low numbers and harvesting restrictions (*Anticlea elegans* (syn. *Zigadenus glaucus*), *Cirsium pitcheri*, *Populus balsamifera*, *Prunus pumila*, and *Salix cordata*). We did not include functional traits for *Ammophila* in our study as we were interested in how treatments impacted colonizing species’ traits (see Walker et al. 2025 for information about treatment effects on *Ammophila* functional traits).

Plants were assessed for the following traits: total biomass, aboveground (AG) biomass, belowground (BG) biomass (including both root and rhizome tissues), root biomass, root length, specific root length (SRL; m of root length/g of root biomass), root tissue density (RTD; g of root biomass/cm^3^ of root volume), and specific leaf area (SLA; one-sided leaf area in cm^2^/g of leaf biomass). These traits were selected because they are indicative of total plant size, relative and absolute investment in different compartments, and the plant economics spectrum (Wright et al. 2004; Weigelt et al. 2021). Root traits were evaluated on all fine roots (≤ 2 mm in diameter) attached to each individual plant sample. Roots were thawed and rehydrated prior to scanning them at 600 dpi on an Epson flat-bed scanner. Root scans were then analyzed for root length and volume using the WinRHIZO software (Regent Instruments 2021) and then dried for 48 h at 65 °C and weighed for dry biomass. Two samples of *Lithospermum* did not have any fine roots at the sampling depth due to their substantial taproot, and for these samples the species average fine root trait values were used. The largest living leaf of each individual plant was removed above the ligule (for grasses) or petiole (for forbs) and scanned at 600 dpi using an Epson flat-bed scanner. Leaf area was determined using the ImageJ software (Schneider et al. 2012) and then leaves were dried for 48 h at 65° C and weighed for dry biomass for SLA calculation. The remaining above- and belowground plant parts were assessed for dry biomass using the same method described above.

### Community trait metrics

From these trait values, mean values for each species were calculated for each trait. Species mean trait values and their abundances within the experiment for each plot and year were then used to calculate community-weighted mean (CWM) traits using the following formula:

$$\:CWM=\sum_{i=0}^{N}{p}_{i}{x}_{i}$$

where *N* is the number of species in a community, *p*_*i*_ is the proportional abundance of species *i*, and *x*_*i*_ is the trait value of species *i* (de Bello et al. 2021a). Weighted means were calculated in R version 4.2.1 using the function weighted.mean() from the stats package (R Core Team 2022). We also measured functional trait diversity in each plot by calculating functional dispersion (FDis) in R using the dbFD() function from the FD package (Laliberté et al. 2014). Functional trait dispersion (FDis) measures the total distance of each species to the abundance-weighted average for the community (i.e., the CWM) in multivariate trait space. In our study, individual FDis values were calculated as the average distances of the centroid for each species present in a plot to the CWM of that plot within multivariate trait space (Laliberté et al. 2014). When species richness is one, FDis is therefore zero, and these instances were excluded from the analysis (*n* = 19). FDis was calculated from a species × species Euclidean distance matrix of the following trait values: total biomass, root length, SRL, RTD, and SLA. Other biomass measurements (aboveground biomass, belowground biomass, and root biomass) were not included in FDis calculations as they are highly correlated with each other, and including correlated traits in functional diversity metrics such as FDis can cause issues including artificial convergence and overinflation of metrics (Cadotte et al. 2011).

### Statistical analyses

We analyzed CWM traits and FDis using general linear mixed models in R version 4.2.1 (R Core Team 2022). We used the glmmTMB() function from the glmmTMB package (Brooks et al. 2017). Fixed effects for all models included *Epichloë* and N enrichment as factor variables coded with treatment contrasts using the contr.treatment() function (reference levels: sham-inoculated and control), Year as an ordered factor coded with polynomial contrasts using the contr.poly() function (linear, quadratic, cubic, and quartic trends—which include all possible trends that could be assessed on a 5-level ordered factor—were included to determine whether there was trend over time and its shape), and their interaction (R Core Team 2022). The three-way interaction was not included for CWM RTD due to model convergence issues, and Year was treated as a non-interacting factor. Interaction plots of the raw data showed variation in the intercepts and trajectories of individual plots over time. To account for this and repeated measurements, we included a random intercept and slope over time for each plot. Models showed no temporal autocorrelation which was tested using the testTemporalAutocorrelation() function from the DHARMa package (Hartig 2024), which performs a Durbin-Watson test on the scaled model residuals. All CWM traits and FDis model residuals, except for CWM SLA, root length, and SRL, showed significant spatial autocorrelation, which was evaluated using the testSpatialAutocorrelation() function, also from the DHARMa package (Hartig 2024), which calculates a global Moran’s *I* among all plots using a Euclidean distance matrix of plot locations. A fixed effect of plot position by row (as a continuous variable) within the experiment was added to these models, which sufficiently accounted for spatial autocorrelation. The inclusion of the plot position by row accounted for a previously documented slope effect within the experiment and accounted for this variation better than a block term (Emery et al. 2015). To test for model overfitting, we used k-folds cross validation on 10 folds using the cv() function from the cv package (Fox and Monette 2024), using mean square error (MSE) as the criterion on the full sample and with bias correction for small sample size. Models were compared simultaneously using identical folds to reduce error (Fox and Monette 2024).

Simulated model residuals were created using the simulateResiduals() function, and evaluated using the testResiduals(), plot(), and testCategorical() functions from the DHARMa package (Hartig 2024), and response variables log-transformed when necessary to improve normality. Variance was allowed to vary among treatments and years using the dispformula argument within glmmTMB() where necessary. Significance (at α = 0.05) of the fixed effects was assessed using the Anova() function from the car package (Fox and Weisberg 2019) on the type II sums of squares, which calculates a Wald’s Chi-square test. When a significant interaction was detected, interacting factor levels were re-coded as the combination of interacting factors for pairwise comparisons. We used the emmeans package (Lenth 2025) to extract estimated marginal means using the emmeans() function and conducted planned contrasts using the contrasts() function. Trend for Year was determined using polynomial contrasts. Orthogonal planned contrasts were used to evaluate interactions between *Epichloë* and N enrichment, which compared control and N enrichment treatments and then N enrichment treatments within each *Epichloë* group. Data were back-transformed and estimated marginal means and standard errors (SEs) were used for mean comparisons and visualization. When a significant interaction between *Epichloë* and N enrichment was detected, we evaluated whether there was an effect of *Ammophila* prevalence using a second model that included log-transformed *Ammophila* abundance as a covariate.

Due to strong non-normality in the residuals of FDis models, we applied a power transformation (x^2^) to the response variable after using the boxcox() function from the MASS package (Venables and Ripley 2002) to determine the best transformation. The model fixed effects and pairwise comparisons were evaluated on the power-transformed response scale and then back-transformed for visualization and reporting as described above. Polynomial contrasts indicated a linear trend over time, so the significant interaction between N enrichment and Year was reassessed using Year as a continuous variable to compare trends among N enrichment treatments. We used emtrends() and contrasts() from the emmeans package to compare linear trends among groups and the test() function to determine whether trends differed from zero slope. Estimated marginal means were then calculated from back-transformed data for visualization purposes as described above.

## Results

### Community-weighted mean (CWM) functional traits

**Fig. 1:**
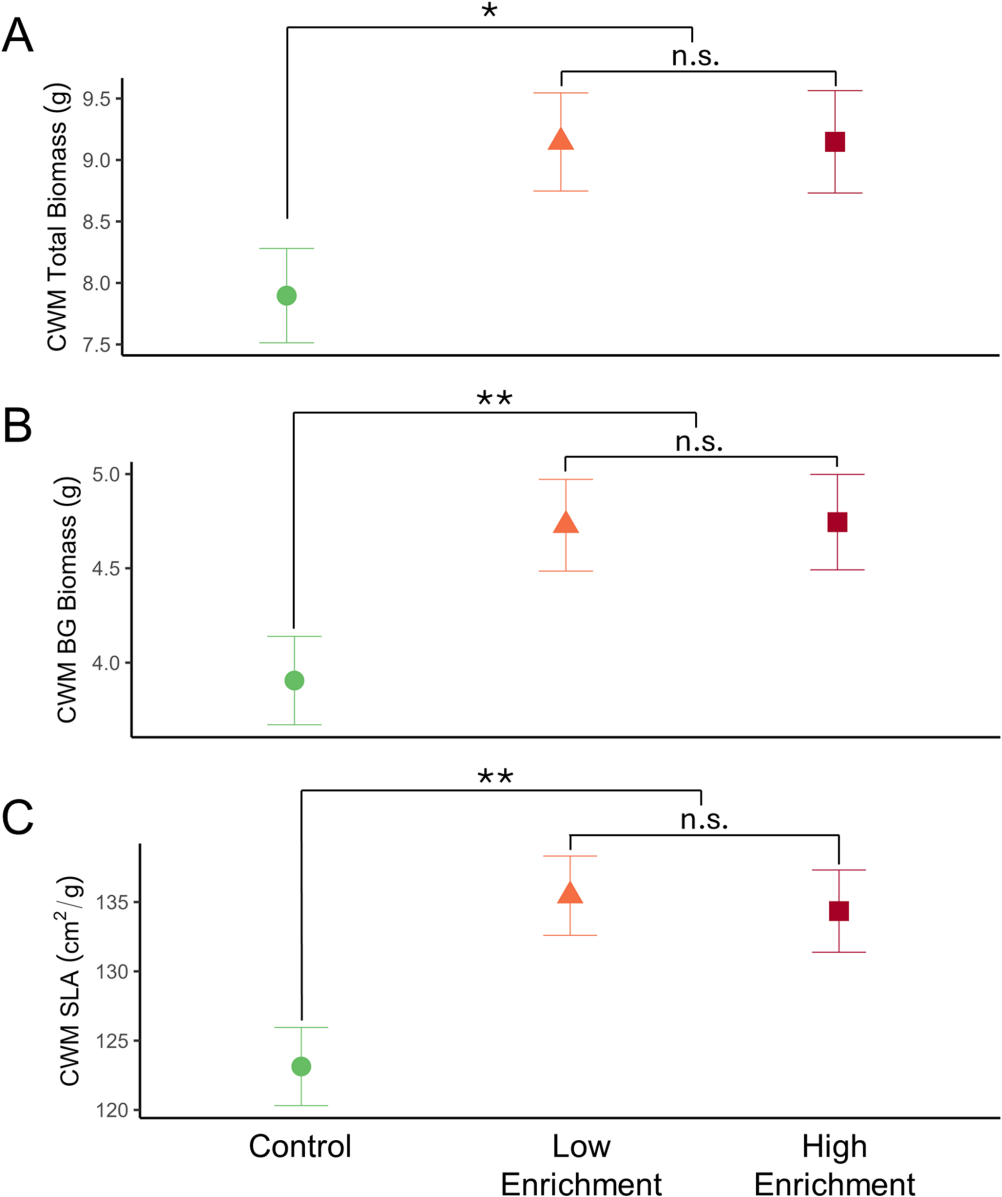
N enrichment effects on select CWM traits. N enrichment increased CWMs of (**A**) total biomass (*p* = 0.01), (**B**) BG biomass (*p* = 0.005), and (C) specific leaf area (SLA; *p* = 0.0009). Data show estimated marginal means and SEs with planned comparisons of the effect of N enrichment treatment versus control (i.e., no treatment) and comparisons of N enrichment levels. Significance levels of planned contrasts based on α = 0.05 are denoted by asterisks (* < 0.05, ** < 0.01, *** <0.001)

N enrichment had significant main effects on CWMs of total biomass, BG biomass, SLA, AG biomass, root biomass, and root length (Fig. 1, Supplementary Fig. 1 and Supplementary Table 1). N enrichment increased measures of SLA and plant size, especially belowground, compared with controls, regardless of treatment. Specifically, N enrichment treatments increased CWMs for total biomass by 7% (*p* = 0.01; Fig. 1A), BG biomass by 18% (*p* = 0.005; Fig. 1B), SLA by 9% (*p* = 0.0009; Fig. 1C), root biomass by 21% (*p* = 0.003; Supplementary Fig. 1B), and root length by 12% (*p* = 0.01; Supplementary Fig. 1C) across both high and low treatments compared with controls. There was a similar trend of increasing CWM of AG biomass, though N enrichment treatments did not differ from controls following planned comparisons (*p* = 0.08; Supplementary Fig. 1A; Supplementary Table 1). Low and high N enrichment treatments did not differ in any planned comparisons, indicating they had similar effects on CWMs.

**Fig. 2:**
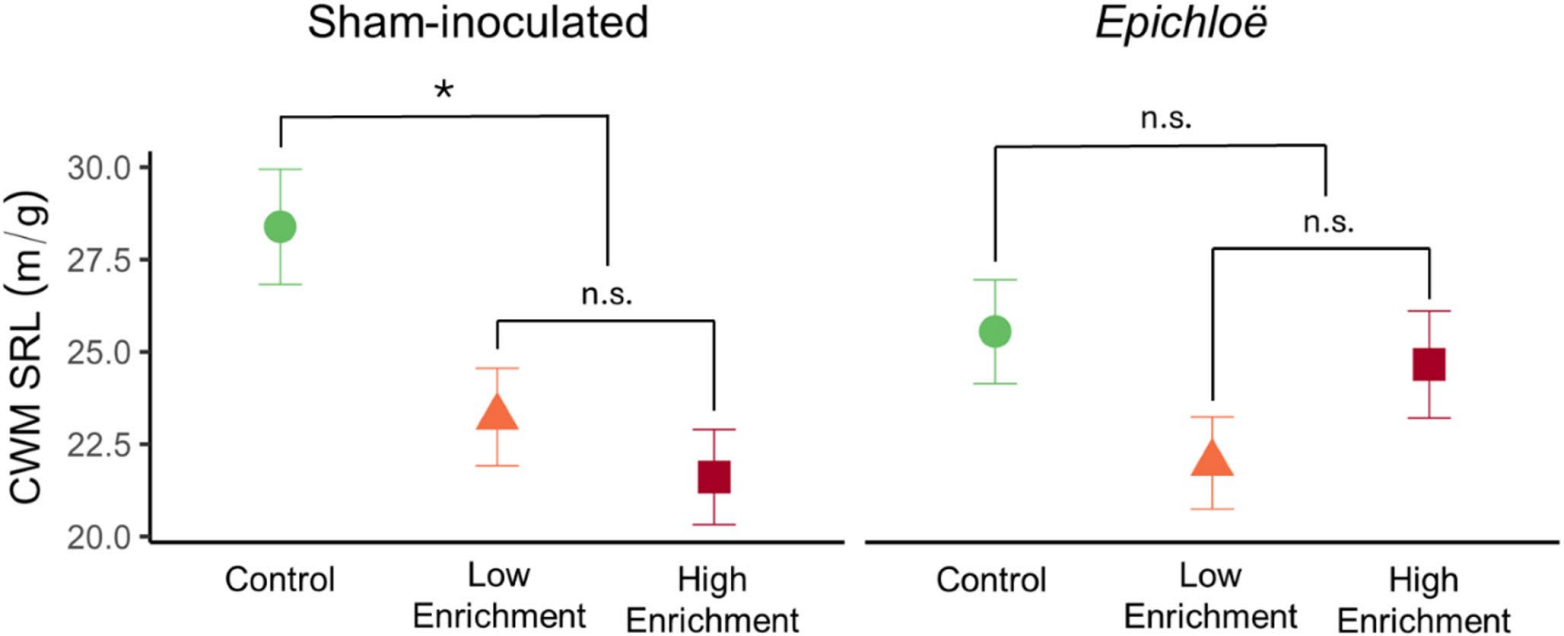
*Epichloë* effects on CWM SRL response to N enrichment. While N enrichment decreased CWM SRL in sham-inoculated plots (*p* = 0.02) compared with controls, *Epichloë* overrode this effect. Data show estimated marginal means and SEs with planned comparisons of the effect of N enrichment treatment versus control (i.e., no treatment) and comparisons of N enrichment levels. Significance levels of planned contrasts based on α = 0.05 are denoted by asterisks (* < 0.05, ** < 0.01, *** <0.001)

While *Epichloë* had no main effects on any measured functional trait (Supplementary Table 1), *Epichloë* x N enrichment had an interactive effect on CWM SRL (X^2^ = 6.93, df = 2, *p* = 0.03; Fig. 2; Supplementary Table 1). N enrichment treatments had significant effects in sham-inoculated plots, while *Epichloë*-inoculated plots did not show any response to different N enrichment levels (Fig. 2). Specifically, N enrichment decreased CWM SRL when *Epichloë* was absent (*p* = 0.02; Fig. 2), with sham-inoculated *Epichloë* plants in low N enrichment having 18% lower and high N enrichment having 24% lower values of SRL than controls. *Ammophila* abundance was not a significant covariate in the CWM SRL model (Supplementary Table 1).

Year had a significant main effect in all CWM traits models (Supplementary Table 1). All CWM traits exhibited a quartic (i.e., x^4^) trend over time, indicating high stochasticity in community traits: total biomass (*p* < 0.0001), AG biomass (*p* < 0.0001), BG biomass (*p* < 0.0001), root biomass (*p* < 0.0001), SLA (*p* < 0.0001), SRL (*p* = 0.009), RTD (*p* < 0.0001), and root length (*p* < 0.0001; Supplementary Fig. 2).

### Functional dispersion (FDis) of Traits

**Fig. 3:**
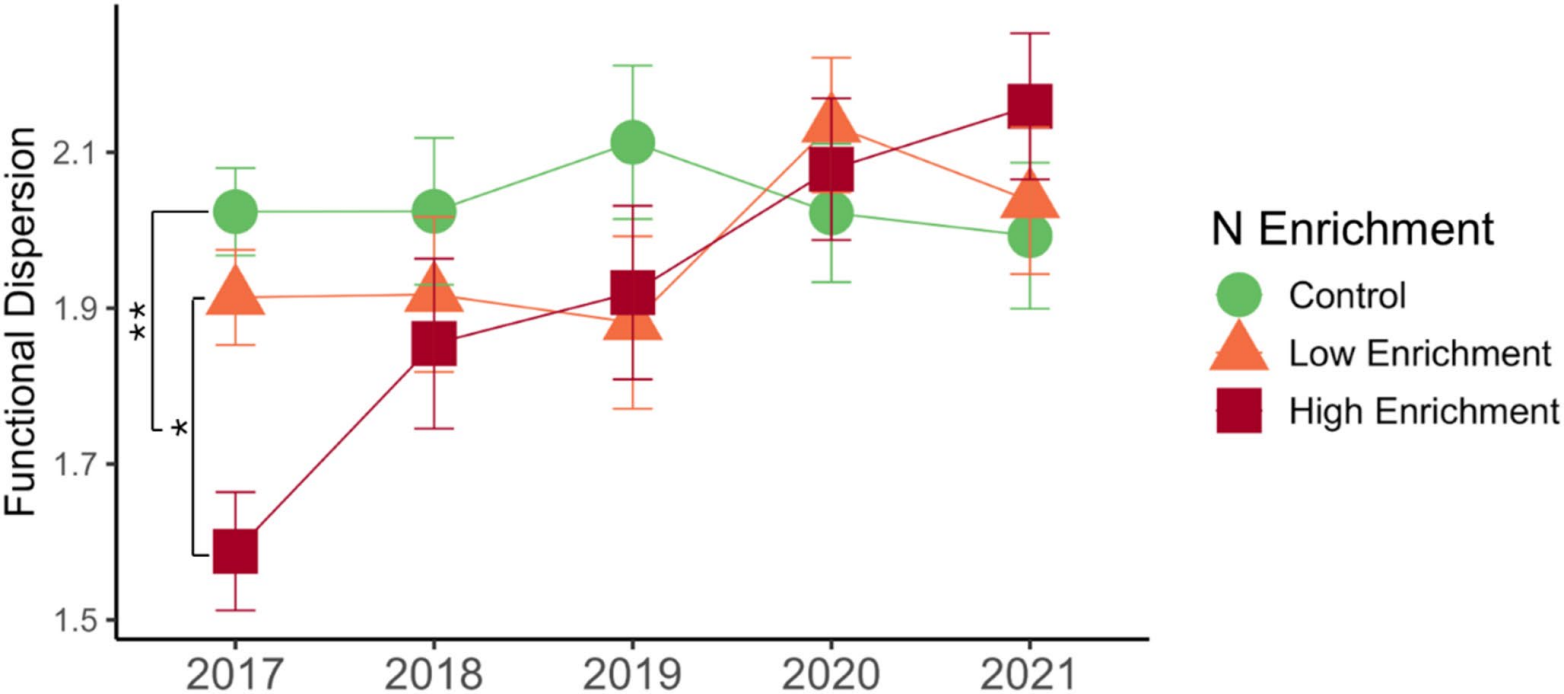
N enrichment effects on functional dispersion (FDis). N enrichment affected functional dispersion (FDis) relative to controls (*p* = 0.008), with trends differing between low and high N enrichment groups (*p* = 0.02). FDis increased linearly over time in high N enrichment plots (slope = 0.54 ± 0.12, *p* < 0.0001). Significance levels of planned contrasts based on α = 0.05 are denoted by asterisks (* < 0.05, ** < 0.01, *** <0.001)

N enrichment treatment and the linear trend of year interactively affected FDis (X^2^ = 15.51, df = 8, *p* = 0.05; linear trend *p* = 0.005; Fig. 3; Supplementary Table 1), with trends differing between N enrichment groups and controls (*p* = 0.008) and between low and high N enrichment groups (*p* = 0.02). High N enrichment groups increased functional dispersion over time (slope = 0.54 ± 0.12, *p* < 0.0001) while slopes for control (slope = -0.02 ± 0.11, *p* = 0.83) and low N enrichment (slope = 0.16 ± 0.12, *p* = 0.18) groups were not significantly different from zero (Fig. 3). Again, highly correlated biomass metrics (aboveground biomass, belowground biomass, and root biomass) were not included in FDis as these can cause issues including artificial convergence and overinflation of metrics (Cadotte et al. 2011). There was no significant effect of *Epichloë* on FDis.

## Discussion

### N enrichment alters trait means and trait diversity

We found that N enrichment increased community-level metrics of plant size (biomass) and specific leaf area (SLA), similar to findings from other grassland systems (e.g., Xu et al. 2018; Guo et al. 2022). Though coastal sand dune communities are adapted to low resource availability and thought to be composed of more resource-conservative, slow-growing plant species (Grime 1977; Chapin et al. 1986; Cleland and Harpole 2010), N enrichment still shifted community-level plant traits and strategies. Further, the effects of low N enrichment (representative of N deposition rates around Chicago, IL, USA) were comparable to those of high N enrichment, indicating that real-world N deposition levels may be sufficient to drive changes in plant trait composition by favoring the growth of larger, more competitive plant species.

Plant communities composed of larger plants with more acquisitive leaves with higher values of SLA typically have greater productivity and more labile tissues which can alter nutrient cycling, C fluxes and sequestration, patterns of herbivory, and soil fertility (de Bello et al. 2010; de Bello et al. 2021b; Hanisch et al. 2020). Additionally, in coastal dune systems, both above- and belowground structures physically interact with wind and wave energies, with productive communities that have higher plant densities attenuating erosive forces and trapping and stabilizing highly mobile sediments more readily (Feagin et al. 2015; de Battisti 2021). While we did not evaluate productivity in this study, shifts in functional traits such as plant size and SLA due to N enrichment are generally associated with greater productivity and plant size metrics (Cleland and Harpole 2010; Xu et al. 2018; Guo et al. 2022). Human-driven inputs of N may therefore increase productivity and alter erosional dynamics even in low-nutrient ecosystems such as coastal sand dunes where plant taxonomic diversity is low and plant species are thought to exhibit more conservative plant growth strategies (Grime 1977). There were no clear directional shifts in community mean traits over time—instead mean traits followed a quadratic trend indicating both high stochasticity and a lack of plant successional effects. The lack of directional temporal pattern may be attributable to low species richness, which could contribute to high temporal instability including in response to environmental perturbations such as drought or disease (Johnson et al. 1996; Isbell et al. 2009).

While both low and high N enrichment shifted community mean traits, trait diversity (FDis) was only significantly affected by high N enrichment. High N enrichment appeared to initially suppress plant functional trait diversity relative to low N and controls and then increased over time. Though there may be positive successional effects on functional trait diversity in high N plots (Fig. 3), these effects could also be attributed to interannual variability or initial application effects. The reduction of overall trait diversity in high N treatments could be due to negative effects on plant diversity as documented in this study and others (Cleland and Harpole 2010; Garces et al. 2023), yet this effect is not necessarily obvious as N enrichment has been shown to have positive or no effects on diversity metrics in other dune systems (e.g., Brown et al. 2022; Garces et al. 2023). The sudden reduction of functional trait diversity may, however, indicate that high levels of N enrichment could initially reduce ecosystem stability by causing the one-time loss of functionally unique species or altering patterns of species dominance as was shown for this system in Garces et al. (2023), which may have implications for the resilience of these already stochastic ecosystems (Johnson et al. 1996; Isbell et al. 2009; Liu et al. 2019; de Bello et al. 2021b). In our study, it is reassuring that trait diversity of the community could rebound after initial reductions in response to high levels of fertilization.

While caution is warranted when attributing shifts in CWM traits to selective responses in environmental conditions, especially in low-diversity systems (Miller et al. 2019; de Bello et al. 2021a), trait responses in plant size and SLA in our study follow with previous investigations (e.g., Xu et al. 2018; Guo et al. 2022), indicating that similar mechanisms are likely. We also did not investigate intraspecific trait variation in this study, thus changes in CWM traits are linked with N enrichment effects on species composition. Garces et al. (2023) investigated N enrichment effects on community composition, finding that N enrichment communities differed from controls, and this was attributable to increasing grass and declining forb abundance. Two grasses that increased in abundance—*Schizachyrium scoparium* and *Calamovilfa longifolia*—are particularly prevalent in this system and have relatively high biomass and SLA (Walker and Emery 2026), and most likely substantially influenced trait patterns in our study. By contextualizing these N-driven community shifts in terms of specific functional traits and strategies—i.e., shifts toward larger plants with faster aboveground growth—however, our findings are more generalizable to other systems (Díaz and Cabido 2001; Suding et al. 2008; Adler et al. 2013; Westoby 2024). Because functional traits are mechanistically linked with ecosystem function via their responsiveness to environmental variation and effects on ecosystem processes (Loreau et al. 2001; Suding et al. 2005; Xu et al. 2018), trait-based approaches may better predict changes in ecosystem function with global change than taxonomic-based metrics such as species identity and richness (Díaz and Cabido 2001; Funk et al. 2017; Xu et al. 2018).

### Fungal symbiosis alters specific root length (SRL) response to N enrichment

N enrichment also reduced SRL CWMs, but only in sham-inoculated plots. SRL is generally thought to measure root foraging capacity, with high SRL species investing in thinner roots that are better able to forage for soil resources (Bergmann et al. 2020; Weigelt et al. 2021). SRL has been shown to decrease with increasing N enrichment due to a decline in fine root and increase in coarse root biomass (Li et al. 2015) and may indicate a shift in belowground strategy away from upper soil root foraging for resources with increased N resource abundance (Bergmann et al. 2020; Weigelt et al. 2021). Such shifts in root system architecture can have direct impacts on belowground processes such as carbon storage, soil stabilization, and nutrient cycling (Bardgett et al. 2014) and indirect effects on these processes via changes in root-associated microbial communities (Sweeney et al. 2021; Nunez-Mir and McCary 2024).

Notably, we found that the presence of *Epichloë* in the dominant host plant overrode the effects of N enrichment on colonizing plant SRL CWMs. While SRL decreased in response to N enrichment in the absence of *Epichloë*, this effect disappeared in the presence of the fungal endophyte. *Epichloë* appeared to suppress this N enrichment effect mainly by slightly reducing community-level SRL in control and increasing it in high N enrichment plots, leading to a convergence of SRL means across years and N treatments. Despite being an aboveground fungal endophyte, *Epichloë* is known to alter soil nutrient cycling and reduce soil microbial diversity (Clay and Schardl 2002; Chen et al. 2022), which may have contributed to these effects on a key root trait. Indeed, previous studies in the long-term experiment have indicated that *Epichloë* may play a pivotal role in structuring belowground communities (Bell-Dereske et al. 2017a; Garces et al. 2022), and changes in soil communities can have both direct and indirect effects on plant communities via numerous pathways such as changes in plant productivity, nutrient cycling, and pathogens (van der Heijden et al. 2008). Another study showed that *Epichloë* increases *Ammophila* litter decomposition, which affected belowground nutrient cycling (Bell-Dereske et al. 2017b). These effects may be due to the production of toxic, N-rich alkaloids (Clay and Schardl 2002; Bell-Dereske et al. 2017a, b; Garces et al. 2022) which may suppress certain fungal symbionts and alter belowground communities via effects on soil N cycling. Lastly, *Epichloë* has been shown to alter *Ammophila* functional traits (Emery et al. 2015; Walker et al. 2025) and biomass (Bell-Dereske et al. 2017b; Walker et al. 2025) which could alter plant communities via changes in competitive dynamics in plots with *Epichloë* beyond endophyte effects on host plant abundance.

While other studies have documented endophyte-driven shifts in plant community taxonomic diversity and dominance (e.g., Marks et al. 1991; Garces et al. 2023), this is the first study, to our knowledge, that has evaluated their effects on community-level functional traits in the presence of a major global change driver. That the presence of *Epichloë* within the dominant plant species impacted a community-level root trait in colonizing plant species is notable, especially since these endophytes may be relatively common in many dominant species of grassland systems (Leuchtmann 1993; Clay and Schardl 2002; Saikkonen et al. 2006). Studies in systems where *Epichloë* host plants are common that do not consider endophyte presence may erroneously attribute endophyte effects to other factors, and more study is needed to understand the contexts within which *Epichloë* fungi may have meaningful impacts. Further, the myriad and substantial effects *Epichloë* can have on host plant traits and key regulators of plant community dynamics (e.g., soil conditions, herbivory) indicate that further study is needed to evaluate the mechanisms and relative importance of their effects.

## Supplementary Information

Below is the link to the electronic supplementary material.

Supplementary Material 1.

## Data Availability

The datasets supporting this study are publicly available on the Zenodo database: 10.5281/zenodo.19433576 (Walker and Emery 2026).

## Code availability

All code used for this study was from standard and publicly available packages. Dynamic documents of R code are publicly available on the Zenodo database: 10.5281/zenodo.19433576 (Walker and Emery 2026).
