# Supplementary material for: Fungal symbiosis alters non-host, community-level plant trait response to N enrichment in a low-nutrient sand dune system
Source: Oecologia. 2026 May 31;208(6):74. doi: 10.1007/s00442-026-05914-5 (PMC13222889; doi:10.1007/s00442-026-05914-5)

Online Resource

Title: Fungal symbiosis alters non-host, community-level plant trait response to N enrichment in a low-nutrient sand dune system

Authors: Shannon L. Walker^1^ (ORCID: 0000-0002-2598-2456), Sarah M. Emery^1^ (ORCID: 0000-0003-2496-6152)

Affiliations

^1^Department of Biological Sciences and Center for Great Lakes and Watershed Studies, Bowling Green State University, Bowling Green, OH 43402, USA

Corresponding Author: Shannon L. Walker

Email: [walkesl@bgsu.edu](mailto:walkesl@bgsu.edu)

Phone: (419) 372-8551

FAX: N/A

**Online Resource Supplementary Tables and Figures**

**Online Resource Supplementary Table 1**. Interaction and main effects for general and generalized linear mixed models of CWM traits and functional dispersion. Light grey text indicates main effects that were not interpreted due to the presence of significant higher-order interactions. Significant interaction and main effects are bolded, and significance levels based on α = 0.05 are denoted by asterisks (* < 0.05, ** < 0.01, *** <0.001). NA values indicate that a given factor/covariate was not included in the model.

| **Community Trait Metric** | **Effect** | **Chi-sq** | **DF** | **p-value** | **Sig. code** |
| --- | --- | --- | --- | --- | --- |
| **CWM Total Biomass** | *Epichloë* x N enrichment | 1.86 | 2 | 0.39 |  |
|  | *Epichloë* x Year | 0.84 | 4 | 0.93 |  |
|  | N enrichment x Year | 6.72 | 8 | 0.57 |  |
|  | *Epichloë* x N enrichment x Year | 8.92 | 8 | 0.35 |  |
|  | *Epichloë* | 0.098 | 1 | 0.75 |  |
|  | **N enrichment** | **9.10** | **2** | **0.01** | ***** |
|  | **Year** | **41.72** | **4** | **<0.0001** | ******* |
|  | row | 3.52 | 1 | 0.06 |  |
| **CWM AG Biomass** | *Epichloë* x N enrichment | 3.86 | 2 | 0.15 |  |
|  | *Epichloë* x Year | 1.10 | 4 | 0.89 |  |
|  | N enrichment x Year | 6.86 | 8 | 0.55 |  |
|  | *Epichloë* x N enrichment x Year | 8.20 | 8 | 0.41 |  |
|  | *Epichloë* | 0.002 | 1 | 0.97 |  |
|  | **N enrichment** | **7.19** | **2** | **0.03** | ***** |
|  | **Year** | **29.60** | **4** | **<0.0001** | ******* |
|  | row | 1.59 | 1 | 0.21 |  |
| **CWM BG Biomass** | *Epichloë* x N enrichment | 1.31 | 2 | 0.52 |  |
|  | *Epichloë* x Year | 0.80 | 4 | 0.94 |  |
|  | N enrichment x Year | 6.59 | 8 | 0.58 |  |
|  | *Epichloë* x N enrichment x Year | 9.37 | 8 | 0.31 |  |
|  | *Epichloë* | 0.15 | 1 | 0.70 |  |
|  | **N enrichment** | **9.68** | **2** | **0.008** | ****** |
|  | **Year** | **42.84** | **4** | **<0.0001** | ******* |
|  | **row** | **4.52** | **1** | **0.03** | ***** |
| **CWM Root Biomass** | *Epichloë* x N enrichment | 1.32 | 2 | 0.52 |  |
|  | *Epichloë* x Year | 0.82 | 4 | 0.94 |  |
|  | N enrichment x Year | 7.79 | 8 | 0.45 |  |
|  | *Epichloë* x N enrichment x Year | 8.80 | 8 | 0.36 |  |
|  | *Epichloë* | 0.25 | 1 | 0.62 |  |
|  | **N enrichment** | **9.51** | **2** | **0.009** | ****** |
|  | **Year** | **42.03** | **4** | **<0.0001** | ******* |
|  | **row** | 3.72 | 1 | 0.05 |  |
|  |  |  |  |  |  |
| **CWM SLA** | *Epichloë* x N enrichment | 0.76 | 2 | 0.68 |  |
|  | *Epichloë* x Year | 1.07 | 4 | 0.90 |  |
|  | N enrichment x Year | 10.43 | 8 | 0.24 |  |
|  | *Epichloë* x N enrichment x Year | 9.25 | 8 | 0.32 |  |
|  | *Epichloë* | 0.06 | 1 | 0.81 |  |
|  | **N enrichment** | **11.54** | **2** | **0.003** | ****** |
|  | **Year** | **46.95** | **4** | **<0.0001** | ******* |
|  | row | NA | NA | NA | NA |
| **CWM Root SRL** | ***Epichloë* x N enrichment** | **6.93** | **2** | **0.03** | ***** |
|  | *Epichloë* x Year | 0.98 | 4 | 0.91 |  |
|  | N enrichment x Year | 11.32 | 8 | 0.18 |  |
|  | *Epichloë* x N enrichment x Year | 5.07 | 8 | 0.75 |  |
|  | *Epichloë* | 0.065 | 1 | 0.80 |  |
|  | N enrichment | 16.36 | 2 | 0.0003 | *** |
|  | **Year** | **22.37** | **4** | **0.0002** | ******* |
|  | row | NA | NA | NA | NA |
| **CWM SRL** | ***Epichloë* x N enrichment** | **6.87** | **2** | **0.03** | ***** |
| **(model 2)** | *Epichloë* x Year | 0.95 | 4 | 0.92 |  |
|  | N enrichment x Year | 10.96 | 8 | 0.20 |  |
|  | *Epichloë* x N enrichment x Year | 5.37 | 8 | 0.72 |  |
|  | *Epichloë* | 0.20 | 1 | 0.65 |  |
|  | N enrichment | 17.46 | 2 | 0.0002 | *** |
|  | **Year** | 14.68 | 4 | 0.005 | ** |
|  | row | NA | NA | NA | NA |
|  | log(*Ammophila* abundance) | 1.57 | 1 | 0.21 |  |
| **CWM RTD** | *Epichloë* x N enrichment | 2.58 | 2 | 0.28 |  |
|  | *Epichloë* x Year | NA | NA | NA | NA |
|  | N enrichment x Year | NA | NA | NA | NA |
|  | *Epichloë* x N enrichment x Year | NA | NA | NA | NA |
|  | *Epichloë* | 0.088 | 1 | 0.77 |  |
|  | N enrichment | 5.34 | 2 | 0.07 |  |
|  | **Year** | **34.47** | **4** | **<0.0001** | ******* |
|  | row | 1.78 | 1 | 0.18 |  |
| **CWM Root Length** | *Epichloë* x N enrichment | 1.00 | 2 | 0.61 |  |
|  | *Epichloë* x Year | 0.82 | 4 | 0.94 |  |
|  | N enrichment x Year | 7.96 | 8 | 0.44 |  |
|  | *Epichloë* x N enrichment x Year | 8.55 | 8 | 0.38 |  |
|  | *Epichloë* | 0.039 | 1 | 0.84 |  |
|  | **N enrichment** | **7.21** | **2** | **0.03** | ***** |
|  | **Year** | **38.74** | **4** | **<0.0001** | ******* |
|  | row | NA | NA | NA | NA |

| **FDis** | *Epichloë* x N enrichment | 0.03 | 2 | 0.98 |  |
| --- | --- | --- | --- | --- | --- |
|  | *Epichloë* x Year | 5.75 | 4 | 0.22 |  |
|  | **N enrichment x Year** | **15.51** | **8** | **0.05** | ***** |
|  | *Epichloë* x N enrichment x Year | 6.93 | 8 | 0.54 |  |
|  | *Epichloë* | 1.60 | 1 | 0.21 |  |
|  | N enrichment | 13.20 | 2 | 0.001 | ** |
|  | Year | 11.37 | 4 | 0.02 | * |
|  | **row** | **4.69** | **1** | **0.03** | ***** |

**Online Resource Supplementary Figure 1**. N enrichment generally increased CWMs of (A) AG biomass (p = 0.03), (B) root biomass (p = 0.009), and (C) root length (p = 0.03; Supplementary Table 1), though planned contrasts showed no differences between N enrichment and control groups for CWM AG biomass (p = 0.08). Data show estimated marginal means and SEs with planned comparisons of the effect of N enrichment treatment versus control (i.e., no treatment) and comparisons of N enrichment levels. Significance levels of planned contrasts based on α = 0.05 are denoted by asterisks (* < 0.05, ** < 0.01, *** <0.001).


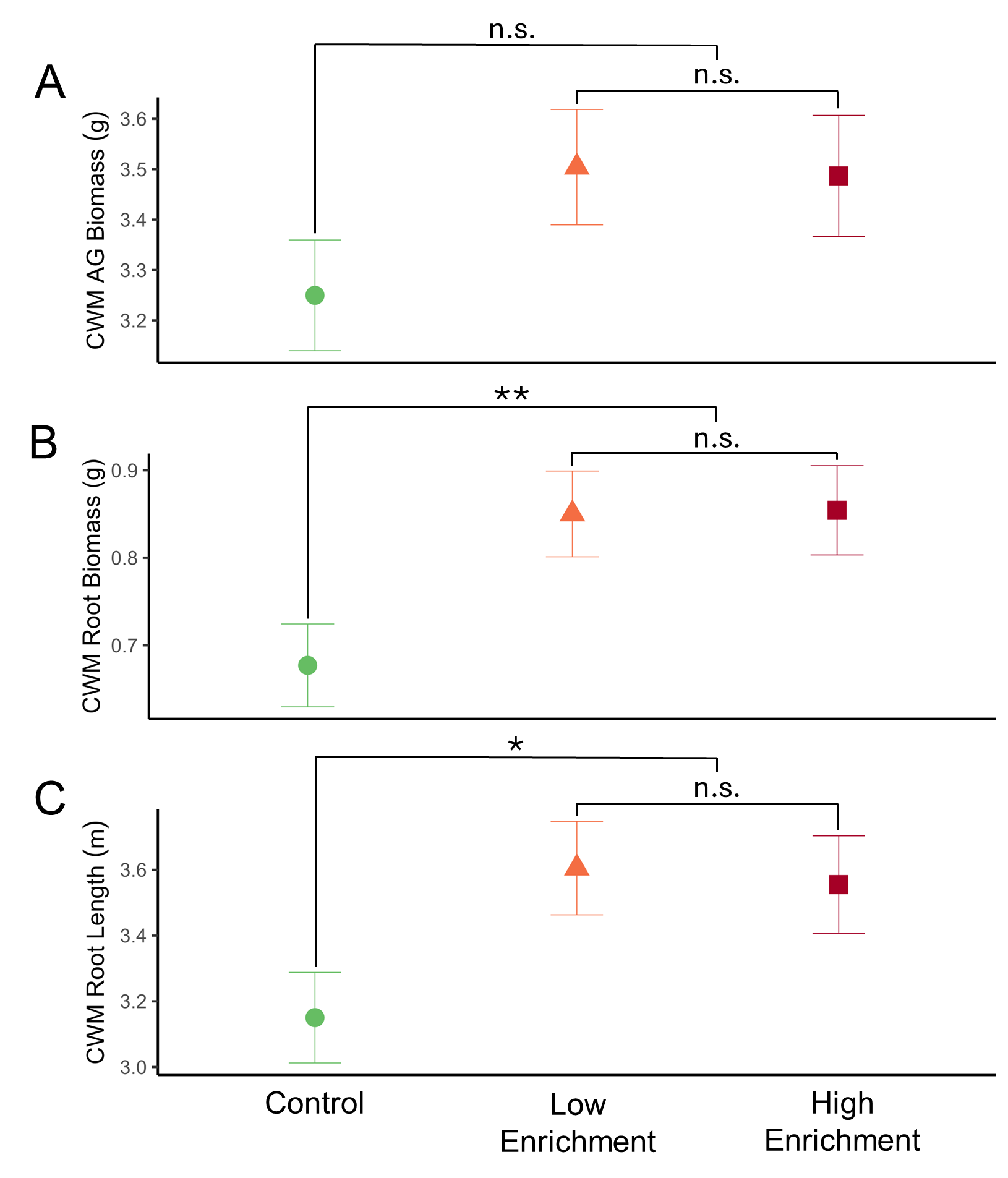


**Online Resource Supplementary Figure 2**. Examples of typical CWM trait trends over time. CWM traits exhibited a quartic trend over time, indicating high stochasticity. Examples of this trend for CWMs of (A) AG biomass (p < 0.0001) and (B) SLA (p < 0.0001) are shown. Data are of estimated marginal means and SEs.


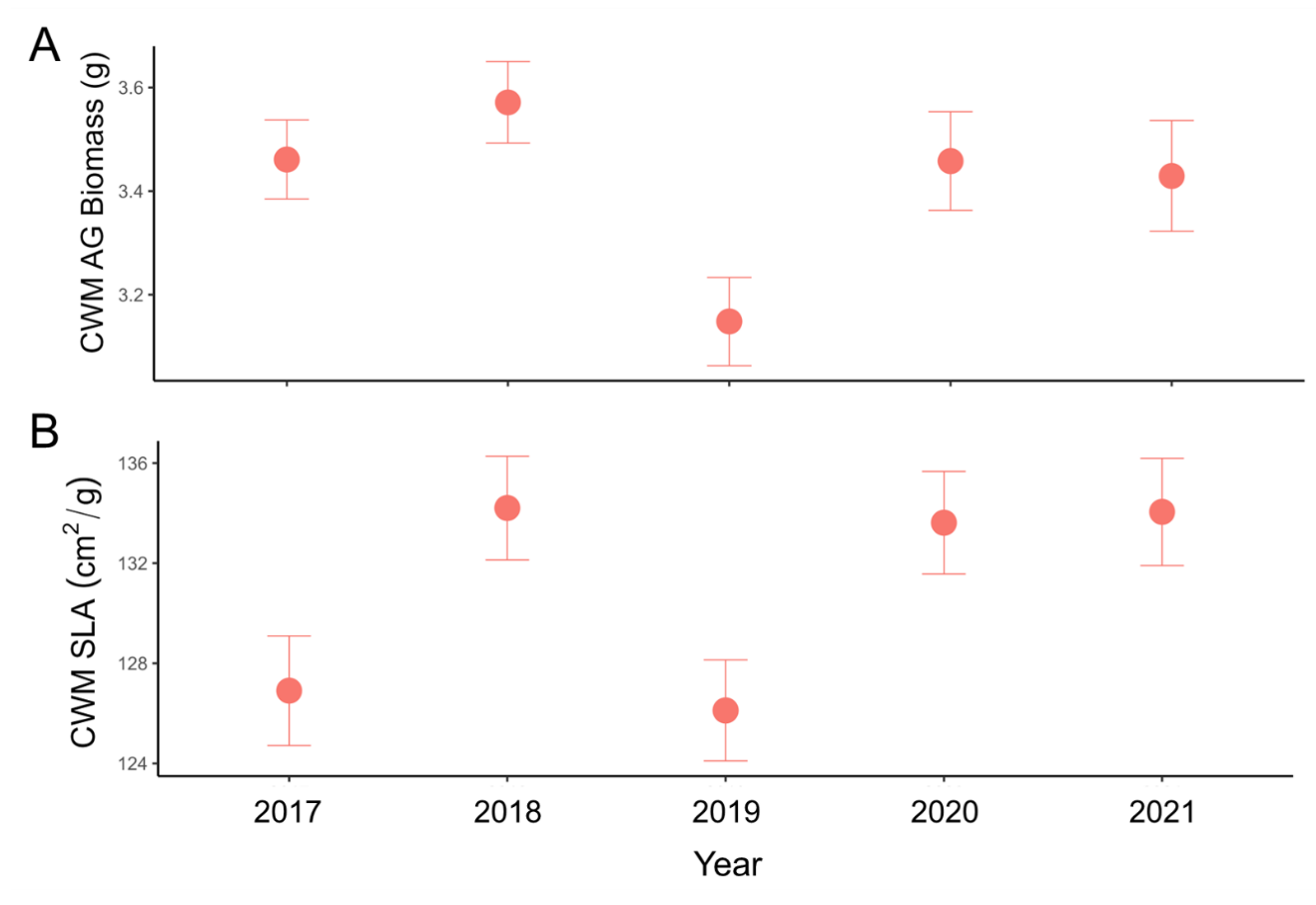

Supplement: Supplementary file 1 — Supplementary Material 1. [file 442_2026_5914_MOESM1_ESM.docx]
